# Nigella Plants – Traditional Uses, Bioactive Phytoconstituents, Preclinical and Clinical Studies

**DOI:** 10.3389/fphar.2021.625386

**Published:** 2021-04-26

**Authors:** Bahare Salehi, Cristina Quispe, Muhammad Imran, Iahtisham Ul-Haq, Jelena Živković, Ibrahim M. Abu-Reidah, Surjit Sen, Yasaman Taheri, Krishnendu Acharya, Hamed Azadi, María del Mar Contreras, Antonio Segura-Carretero, Dima Mnayer, Gautam Sethi, Miquel Martorell, Ahmad Faizal Abdull Razis, Usman Sunusi, Ramla Muhammad Kamal, Hafiz Ansar Rasul Suleria, Javad Sharifi-Rad

**Affiliations:** ^1^ Medical Ethics and Law Research Center, Shahid Beheshti University of Medical Sciences, Tehran, Iran; ^2^ Facultad de Ciencias de la Salud, Universidad Arturo Prat, Iquique, Chile; ^3^ Faculty of Allied Health Sciences, University Institute of Diet and Nutritional Sciences, The University of Lahore, Lahore, Pakistan; ^4^ Department of Diet and Nutritional Sciences, Faculty of Health and Allied Sciences, Imperial College of Business Studies, Lahore, Pakistan; ^5^ Institute for Medicinal Plants Research “Dr. Josif Pančić”, Belgrade, Serbia; ^6^ Department of Environmental Science/Boreal Ecosystem Research Initiative, Memorial University of Newfoundland, Corner Brook, NL, Canada; ^7^ Molecular and Applied Mycology and Plant Pathology Laboratory, Department of Botany, University of Calcutta, Kolkata, India; ^8^ Department of Botany, Fakir Chand College, Diamond Harbour, India; ^9^ Phytochemistry Research Center, Shahid Beheshti University of Medical Sciences, Tehran, Iran; ^10^ Department of Agronomy and Plant Breeding Science, College of Aburaihan, University of Tehran, Tehran, Iran; ^11^ Department of Chemical, Environmental and Materials Engineering, University of Jaén, Jaén, Spain; ^12^ Department of Analytical Chemistry, Faculty of Sciences, University of Granada, Granada, Spain; ^13^ Research and Development Functional Food Centre (CIDAF), Bioregión Building, Health Science Technological Park, Granada, Spain; ^14^ Faculty of Sciences, Lebanese University, Beirut, Lebanon; ^15^ Department of Pharmacology, Yong Loo Lin School of Medicine, National University of Singapore, Singapore, Singapore; ^16^ Department of Nutrition and Dietetics, Faculty of Pharmacy, and Centre for Healthy Living, University of Concepción, Concepción, Chile; ^17^ Unidad de Desarrollo Tecnológico, UDT, Universidad de Concepción, Concepción, Chile; ^18^ Department of Food Science, Faculty of Food Science and Technology, Universiti Putra Malaysia, Serdang, Malaysia; ^19^ Natural Medicines and Products Research Laboratory, Institute of Bioscience, Universiti Putra Malaysia, Serdang, Malaysia; ^20^ Department of Biochemistry, Bayero University Kano, Kano, Nigeria; ^21^ Department of Pharmacology, Federal University Dutse, Dutse, Nigeria; ^22^ Department of Agriculture and Food Systems, The University of Melbourne, Melbourne, VIC, Australia; ^23^ Facultad de Medicina, Universidad del Azuay, Cuenca, Ecuador

**Keywords:** *Nigella*, cancer, pharmacological properties, functional ingredients, metabolic syndrome, thymoquinone

## Abstract

*Nigella* is a small genus of the family Ranunculaceae, which includes some popular species due to their culinary and medicinal properties, especially in Eastern Europe, Middle East, Western, and Central Asia. Therefore, this review covers the traditional uses and phytochemical composition of *Nigella* and, in particular, *Nigella sativa*. The pharmacological studies reported *in vitro*, *in vivo,* and in humans have also been reviewed. One of the main strength of the use of *Nigella* is that the seeds are rich in the omega-6 fatty acid linoleic acid and provide an extra-source of dietary phytochemicals, including the bioactive thymoquinone, and characteristics saponins, alkaloids, and flavonoids. Among *Nigella* species, *N*. *sativa* L. is the most studied plant from the genus. Due to the phytochemical composition and pharmacological properties, the seed and seed oil from this plant can be considered as good candidates to formulate functional ingredients on the basis of folklore and scientific knowledge. Nonetheless, the main limations are that more studies, especially, clinical trials are required to standardize the results, e.g. to establish active molecules, dosage, chemical profile, long-term effects and impact of cooking/incorporation into foods.

## Introduction

*Nigella*, also known as fennel flower, is a small genus belonging to the family Ranunculaceae and includes around 20 species (Zohary, 1983; The plant list, 2020). The members of the genus are annuals and survive harsh condition as seed (therophytes) with a short life cycle (Agradi et al., 2002). A popular ornamental species, *Nigella damascena* L. (commonly known as lady-in-a-mist or ragged lady), and a well-known condiment and spice, *Nigella sativa* L. (also known as black cumin or black seeds), have a high commercial interest especially in the food, pharmaceutical and cosmetics industries (Ali and Blunden, 2003; Bittkau and Comes, 2009; Malhotra, 2012). As an example, *N*. *sativa* is used in foods, pickles, and baked goods (Malhotra, 2012).

The evolutionary origins of *Nigella* species are presumably in the Aegean and the adjacent Western-Irano-Turanian region; its centre of species diversity (Zohary, 1983; Bittkau and Comes, 2009). The genus is found as wild in southern Europe, Russia, northern Africa, Asia Minor, Turkey, Middle-East, India, Pakistan, and Bangladesh (Tonçer and Kizil, 2004; Zohary, 1983; Dönmez and Mutlu, 2004; Heiss and Oeggl, 2005; Sharma et al., 2009; Iqbal et al., 2010; Heiss et al., 2011; Rabbani et al., 2011; Zhao et al., 2013; Hossan et al., 2018).

The taxonomic position of *Nigella* has undergone many changes in the past few decades. It has been commonly divided into three sections viz. *Komaroffia*, *Garidella* and *Nigella* (Tutin et al., 1972; Tamura, 1993; Uğurlu Aydın and Dönmez, 2019). Section Komaroffia comprises one species (*Nigella integrifolia* Regel), section *Garidella* consists of two species (*Nigella nigellastrum* (L.) Willk. and *Nigella unguicularis* (Poir.) Spenn.), whereas section *Nigella* composed of twelve species, including *N. sativa, N. damascena* and others (*Nigella arvensis* L., *Nigella fumariifola* Kotschy, *Nigella hispanica* L., *Nigella segetalis* M. Bieb., *Nigella stellaris* Boiss., *Nigella elata* Boiss., *Nigella ciliaris* DC., *Nigella orientalis* L., *Nigella oxypetala* Boiss., and *Nigella turcica* Dönmez and Mutlu) (Zohary, 1983; Heiss et al., 2011). In the Plant List, a working list of known plant species produced by the botanical community, there are 91 *Nigella* names, but only 23 are accepted latin names for species (The plant list, 2020). In general, this genus is characterized by angular or discoid seeds and the characteristic black color is related to other common name of *Nigella*, “black cumin” (Zhao et al., 2013).

Due to their ethnopharmacology as healing herbs and food importance of *Nigella* spp., the present work reviews their traditional uses and phytochemical composition*,* as well as various scientific studies related to their health benefit with special emphasis in *N. sativa*.

## Traditional Uses of Nigella

Latest ethno-pharmacological studies showed that *Nigella* species are among the most usually used for traditional and folk medicinal practices. Among them, *N. sativa* is probably the best-known species of the *Nigella* genus and it has been used in many parts of the world as a natural medicine. The traditional use of *N. sativa* dates from the 1st century A.D.; Pliny the Elder recommended *N. sativa* as a digestive and an ingredient of antidotes curing snake bites and scorpion stings (Dönmez and Mutlu, 2004), or even at least till Tutankhamen kingdom (Mrozek-Wilczkiewicz et al., 2016). Today, the seed powder of *N. sativa* is recommended at 0.5–4 g in the Pharmacopoeia of India (Tajmiri et al., 2016), which is used as a stimulant to ease bowel and indigestion problems and as carminative. It has also been administered to manage pain during menstruation and diabetes in India and Bangladesh (Esakkimuthu et al., 2016; Hossan et al., 2018). Similarly, *N. sativa* is widely used in traditional medicine of Algeria for the treatment of diabetes and also to treat high blood pressure (BP) (Bouzabata, 2013).

Moreover, according to the Bedouins (Egypt), the wooden stem is used to treat jaundice, while seeds are used to treat BP as before, as well as heart diseases, etc. (El-Seedi et al., 2013) (Table 1). Similar uses have been reported in Iranian traditional medicine (Ghazeeri et al., 2012; Amiri and Joharchi, 2013; Bahmani et al., 2016b), in Pakistan (Khan et al., 2014; Yaseen et al., 2015; Aziz et al., 2017) and in Morocco (Eddouks et al., 2002; El-Hilaly et al., 2003; Khabbach et al., 2012; Jamila and Mostafa, 2014; Teixidor-Toneu et al., 2016), where *N. sativa* seeds and leaves are orally ingested, consumed as a powder, herbal tea, as decoction or as inhalant. In Pakistan *N. sativa* is also applied to manage lactation, bacterial diseases, etc. (Khan et al., 2014; Aziz et al., 2017), while in Morocco the seeds are recommended to deal with otolaryngological, urological, and nephrological ailments as well as to treat pathologies of the respiratory and skeleton–muscular systems, allergy and hyper-sensibility (Eddouks et al., 2002; El-Seedi et al., 2013; Jamila and Mostafa, 2014). The anti-rheumatic and analgesic properties of *N. sativa* combined with honey have been reported (Khabbach et al., 2012). In addition, seeds infusion is used to treat malaria in the Malaysian traditional medicine (Al-Adhroey et al., 2010), as well as seeds (fresh, dried, and powdered forms) and leaves in Ethiopia (Alrawi et al., 2017). The use of *N. sativa* is still more widespread, being recognized as panacea for its healing properties in Qatar (Alrawi et al., 2017) and Bangladesh (Jennings et al., 2015). Some of the latter uses are also common in Mauritius, Nepal, Turkey, Thailand, Lebanon, and Palestine (Ghazeeri et al., 2012; Al-Ramahi et al., 2013; Sreekeesoon and Mahomoodally, 2014; Guler et al., 2015; Jennings et al., 2015; Kunwar et al., 2015; Bahmani et al., 2016a; Neamsuvan et al., 2016; Alrawi et al., 2017; Ahmed et al., 2018). Furthermore, in Bangladesh and Libano *N. sativa* seeds are used as a spice and food preservative, directly consumed after being ground, while *Nigella* oil also can be applied topically (Jennings et al., 2015). Similarly, seeds of *N. sativa* (also named *Nigella glandulifera* Freyn and Sint.) are consumed in some regions of China and frequently added to “naan” (a crusty pancake). Its water decoction is used in the Uighur's traditional medicine for the treatment of numerous disorders as the other species (Table 1) (Zhao et al., 2013).

**TABLE 1:** Some traditional uses of *Nigella* species.

Species	Traditional use	Country	References
**Asia**			
*N. arvensis* (seed)	To treat lung, brain and skin	Palestine	(Jaradat et al., 2016a)
*N. ciliaris* (seed)	To treat abdominal pain and to facilitate delivery	Palestine	(Ali-Shtayeh et al., 2015)
*N. ciliaris* (seed)	To treat menstrual cycle problems	Iran	(Bahmani et al., 2015)
*N. ciliaris* (seed)	To treat cancer	Palestine	((Jaradat et al., 2016a; Hamarsheh et al., 2017)
*N. sativa* (seed)	To treat diuretic, analgesic, insomnia, dizziness, tinnitus, amnesia, and bronchial disorders	China	(Zhao et al., 2013)
*N. sativa* (seed)	To ease bowel and indigestion problems and to manage diabetes	India	(Tajmiri et al., 2016) (Esakkimuthu et al., 2016)
*N. sativa* (seed)	To manage pain during menstruation and diabetes	Bangladesh	(Esakkimuthu et al., 2016) (Esakkimuthu et al., 2016) (Hossan et al., 2018
*N. sativa* (seed)	Curative effects in bacterial-caused diseases, sexual tonic, to manage lactation and to decrease mental disturbances	Pakistan	(Khan et al., 2014; Aziz et al., 2017)
*N. sativa* (seed)	To treat malaria	Malaysia	(Al-Adhroey et al., 2010)
**Africa**			
*N. sativa* (seed)	Hypoglycemic and hypotensive agent	Algeria	(Bouzabata, 2013)
*N. sativa* (wooden stem and seed)	Wooden steem: To treat jaundice. Seeds: Hypotensive agent and to treat heart diseases, headaches, nasal congestion, toothache, and against intestinal worms	Egypt	(El-Seedi et al., 2013)
*N. sativa* (seed, fruit and leaf)	Hypoglycemic and hypotensive agent and to deal with digestive, respiratory, and cardiovascular problems, and allergy	Morocco	(Eddouks et al., 2002; El-Seedi et al., 2013; Jamila and Mostafa, 2014)
**Europe**			
*N. damascena*	Galactagogue (seed) and against trachoma	Italy	(Geraci et al., 2018) (Leporatti and Ghedira, 2009)
*N. damascena*	Antihelmintic (for children) and to treat haematuria and skin diseases (itchiness and eczema)	Serbia	(Geraci et al., 2018)

Other *Nigella* species with a wide range of medicinal properties are *N. damascena* and *N. ciliaris*. In Central Europe, the use of *N. damascena* dates from Bronze Age but it cannot reliably assign any ethnobotanical relevance and its origin is unclear; it has never grown in the wild in central Europe (Heiss and Oeggl, 2005). Alternatively, *N. damascena* seeds are used for example in Sicilian folk medicine as a galactogogue (Geraci et al., 2018). Other uses are as emmenagogue, vermifugue, and disinfectant (Heiss and Oeggl, 2005). This plant is also used as an helmintic agent and to treat hematuria, and skin diseases in the Serbian medieval medicine (Jarić et al., 2014). The former use has also been reported in Epirus (Greece) (Vokou et al., 1993). Traditionally, *N. damascena* is used for treating trachoma in Tunisia and Italy (Leporatti and Ghedira, 2009). Besides its use as herbal remedy, *N. damascena* is used as a condiment in several regions (Heiss and Oeggl, 2005), including in Morocco (Khabbach et al., 2011).

In the folklore medicine of Palestine and Iran, *N. ciliaris* seeds are used for abdominal pain, to facilitate delivery and to treat menstrual cycle related problems, respectively (Ali-Shtayeh et al., 2015) (Bahmani et al., 2015). In Turkey, Meriç Town, dried flowers of *N. arvensis* are used as a winter tea (Kartal and Güneş, 2017). On the other hand, in Iran (Kerman), the seed powder of *N. arvensis* mixed with other seeds are administrated to enhance male potency and to improve memory and intelligence (Khajoei Nasab and Khosravi, 2014). The seeds of both *Nigella* species are used for the treatment of cancer in Palestine (Jaradat et al., 2016a; Hamarsheh et al., 2017).

## Phytoconstituents

*Nigella* genus is widely used for their culinary and medicinal properties especially in the Eastern Europe, Middle East, Western and Central Asia. The plants are mainly consumed for their seeds and seed oil, as commented before. The main constituents of *N. sativa* seeds reported in the literature are fixed oil (27–40%), proteins (16–19%), characterized mainly by the amino acids arginine, glutamic acid, leucine, lysine; minerals (1.79–3.74%), like Cu, Zn, P, and Fe; carbohydrates (28.5–33.7%), and solubre dietary fibers (5.5–8.9%) (Al-Naqeep et al., 2009; Tiruppur Venkatachallam et al., 2010; Kooti et al., 2016; El-Naggar et al., 2017; Saxena et al., 2017). Concerning the phytochemical composition of *Nigella* seeds, the most interesting plant part, it is constituted of alkaloids, terpenes and phenolic compounds (Agradi et al., 2001). Although the number of studies on the chemical composition and pharmacological properties of *N. sativa* is continuously increasing (Bourgou et al., 2010b), there are other interesting species for their culinary and medicinal properties, as commented before. Thus, this section details phytochemicals found in *N. sativa* and other *Nigella* plants.

### Fixed Oil: Essential Fatty Acids

**FIGURE 1:**
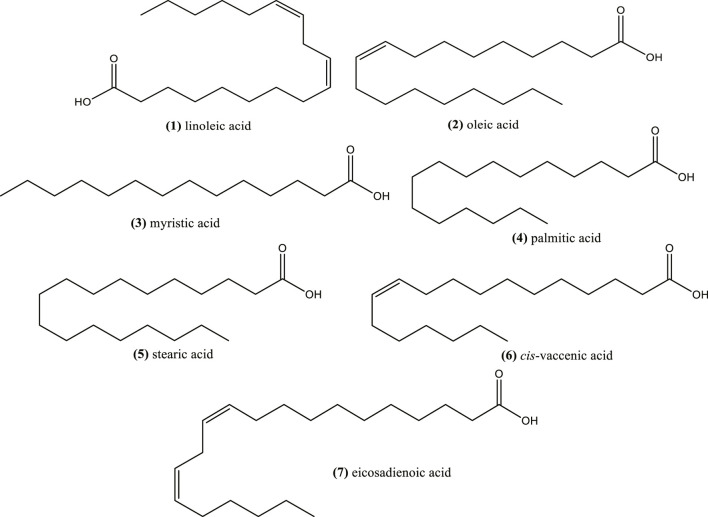
Fatty acids in fixed oils from *Nigella* seeds.

The oil of *N. sativa* seeds has been highly studied, showing predominance of linoleic acid (1) (50–60%), oleic acid (2) (20%), myristic acid (3) (30%), and palmitic acid (4) (12.5%) (Tiruppur Venkatachallam et al., 2010; Kooti et al., 2016; El-Naggar et al., 2017; Saxena et al., 2017) (Figure 1). *Nigella* seed oil from Morocco also showed that the major fatty acids were linoleic acid (1) (58.5 and 56.5%), oleic acid (2) (23.8 and 24.9%) and palmitic acid (4) (13.1 and 11.9%) using cold press and solvent extraction, respectively (Gharby et al., 2015). The major compounds were similar to those found in *N. sativa* seeds from other Mediterranean countries. These fatty acids were also reported as major compounds from the seed oils, extracted by *n*-hexane, of different *N. sativa* genotypes from India (Saxena et al., 2017). Other study have shown that fixed oils from *N. sativa* seed from Turkey and Egypt obtained by supercritical CO_2_ extraction were similar in fatty acid composition of saturated fatty acids (16%), mainly constituted by palmitic acid (4) and stearic acid (5); monounsaturated acids (23%), mainly oleic acid and *cis*-vaccenic acid (6), and polyunsaturated acids (58%), mainly linoleic acid (1) and eicosadienoic acid (7) (Piras et al., 2013) (Figure 1). In this work the ratio omega-6/omega-3 ranged between 180 and 221 considering linoleic and alpha-linolenic acid.

Other *Nigella* species, including *N. damascena*, *N. orientalis*, *N. arvensis*, *N. elata*, *N. nigellastrum*, *N. oxypetala*, *N. segetalis*, *N. unguicularis*, and *N. lancifolia* Hub.-Mor., also contain linoleic acid (31.2–69.5%) and oleic acid (15.8–36.0%) as the major ones (Kökdil et al., 2005; Matthaus and Özcan, 2011). The abundance relevance of other fatty acids depends on the species.

### Volatile Oil Phytochemicals

Volatile oils are derived from plant tissues and are characterized to evaporate under room temperature and failure to saponify. Concerning volatile oil (around 0.5–1.5%) of *Nigella* seeds, it showed high abundance of thymoquinone (8), *p*-cymene (9) and other phenolic derivatives, such as dithymoquinone (nigellone) (10), thymohydroquinone (11), carvacrol (12), and thymol (13) (Bourgou et al., 2010b; Tiruppur Venkatachallam et al., 2010; Kooti et al., 2016; El-Naggar et al., 2017).

**FIGURE 2:**
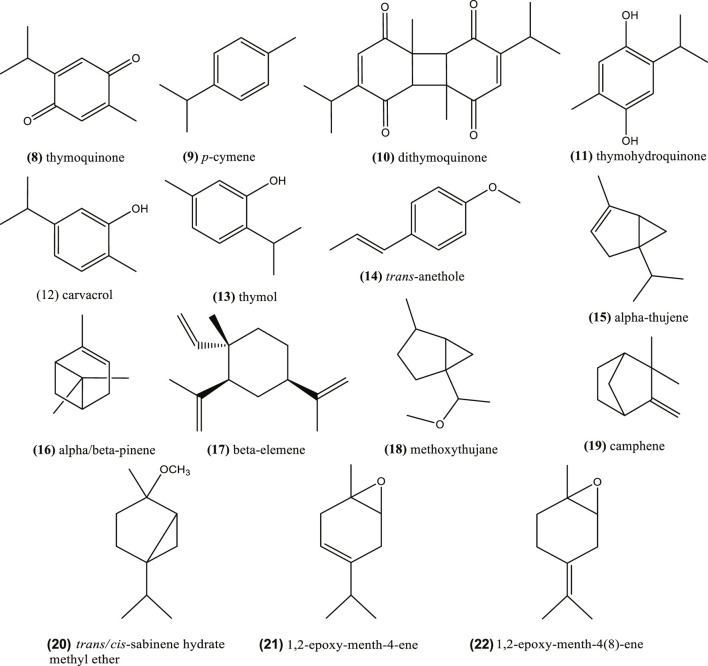
Phytochemical components in essential oils from *Nigella* seeds.

These compounds are considered *Nigella* active principles (Mahmoudvand et al., 2014), but the composition may vary depending on the chemotype and species, among other factors (Edris, 2010; Zribi et al., 2014; Koshak et al., 2017; Saxena et al., 2017). As an example, *N. sativa* volatile composition (seeds) presents different chemotypes (Burits and Bucar, 2000; Islam et al., 2004): thymoquinone (8) chemotype in Egypt and Turkey varieties (77.2–86.2%) (Piras et al., 2013); *trans*-anethole (14) (38.3%) chemotype in Iranian *N. sativa* essential oil (Nickavar et al., 2003); *p*-cymene (9) (33%), and thymol (13) (26.8%) chemotype in Moroccan species (Moretti et al., 2004). The volatile oils from *N. sativa* seeds of Turkey and Egypt obtained by supercritical fractioned extraction with CO_2_ also showed that thymoquinone was the major constituent (77.2–86.2%) followed by *p*-cymene (9) (5.4–11.0%) (Piras et al., 2013). An essential oil from Iranian *N. sativa* seeds was rich in thymoquinone (42.4%), *p*-cymene (14.1%) and caravacrol (10.3%) (Mahmoudvand et al., 2014). Figure 2 depicts the chemical structure of these phytochemicals and Table 2 the content of the major ones and the source.

**TABLE 2:** Main volatile compounds in essential oils from *Nigella* seeds.

Compound name	Source (origin)	Relative amount (%)	References
Thymoquinone	*N. damascena* (M), *N. sativa* seed (C), *N. sativa* seed (T), *N. sativa* seed (I), *N. sativa* seed (tk, E), *N. sativa* seed (ir), *N. sativa* seed (T), *N. sativa* seed (M)	0.1, 3.7, 3.0, 33.1–38.4^a^, 77.2–86.2^b^, 0.6, ND, 3.8	(Bourgou et al., 2010b; Geng et al., 2009; Jrah Harzallah et al. (2011); Moretti et al., 2004; (Piras et al., 2013)
*p*-Cymene	*N. damascena* (M), *N. sativa* seed (C), *N. sativa* seed (T), *N. sativa* seed (I), *N. sativa* seed (tk, E), *N. sativa* seed (ir), *N. sativa* seed (T), *N. sativa* seed (M)	ND, 33.8, 60.5, ND, ND, 14.8, 49.5, 33.8	(Bourgou et al., 2010b; Geng et al., 2009; Jrah Harzallah et al. (2011); Moretti et al., 2004; (Piras et al., 2013)
Dithymoquinone	*N. damascena* (M), *N. sativa* seed (T), *N. sativa* seed (I), *N. sativa* seed (tk, E), *N. sativa* seed (ir), *N. sativa* seed (T), *N. sativa* seed (M)	ND, ND, ND, ND, ND, ND	(Bourgou et al., 2010b; Jrah Harzallah et al. (2011); Moretti et al., 2004; (Piras et al., 2013)
Thymohydroquinone	*N. damascena* (M), *N. sativa* seed (T), *N. sativa* seed (I), *N. sativa* seed (tk, E), *N. sativa* seed (ir), *N. sativa* seed (T), *N. sativa* seed (M)	ND, 0.4, 1.1–2.3^a^, ND, ND, ND	(Bourgou et al., 2010b; Jrah Harzallah et al. (2011); Moretti et al., 2004; (Piras et al., 2013)
Carvacrol	*N. damascena* (M), *N. sativa* seed (T), *N. sativa* seed (I), *N. sativa* seed (tk, E), *N. sativa* seed (ir), *N. sativa* seed (T), *N. sativa* seed (M)	ND, 2.4, 0.8–2.0^a^, ND-7.9, 1.6, 0.6, ND	(Bourgou et al., 2010b; Jrah Harzallah et al. (2011); Moretti et al., 2004; (Piras et al., 2013)
Thymol	*N. damascena* (M), *N. sativa* seed (T), *N. sativa* seed (I), *N. sativa* seed (tk, E), *N. sativa* seed (ir), *N. sativa* seed (T), *N. sativa* seed (M)	ND, ND, 5.3–17.0^a^, ND, ND, 26.8	(Bourgou et al., 2010b; Jrah Harzallah et al. (2011); Moretti et al., 2004; (Piras et al., 2013)
α-Thujene	*N. damascena* (M), *N. sativa* seed (T), *N. sativa* seed (I), *N. sativa* seed (tk, E), *N. sativa* seed (ir), *N. sativa* seed (T), *N. sativa* seed (M)	ND, 6.9, ND, ND-0.4^b^, 2.4, 18.9, 3.3	(Bourgou et al., 2010b; Jrah Harzallah et al. (2011); Moretti et al., 2004; (Piras et al., 2013)
α-Pinene	*N. damascena* (M), *N. sativa* seed (T), *N. sativa* seed (I), *N. sativa* seed (tk, E), *N. sativa* seed (ir), *N. sativa* seed (T), *N. sativa* seed (M)	ND, 1.7, ND, ND, 1.2, 1.2, 5.4, 0.7	(Bourgou et al., 2010b; Jrah Harzallah et al. (2011); Moretti et al., 2004; (Piras et al., 2013)
β-Pinene	*N. damascena* (M), *N. sativa* seed (T), *N. sativa* seed (I), *N. sativa* seed (tk, E), *N. sativa* seed (ir), *N. sativa* seed (T), *N. sativa* seed (M)	ND, 2.4, ND-0.4^a^, ND, 1.3, 4.3, 1.1	(Bourgou et al., 2010b; Jrah Harzallah et al. (2011); Moretti et al., 2004; (Piras et al., 2013)
γ-Terpinene	*N. damascena* (M), *N. sativa* seed (T), *N. sativa* seed (I), *N. sativa* seed (tk, E), *N. sativa* seed (ir), *N. sativa* seed (T), *N. sativa* seed (M)	ND, 3.5, 12.9–27.5^a^, ND-0.6^b^, 0.5, 2.5, 2.4	(Bourgou et al., 2010b; Jrah Harzallah et al. (2011); Moretti et al., 2004; (Piras et al., 2013)
*trans*-Anethole	*N. damascena* (M), *N. sativa* seed (T), *N. sativa* seed (I), *N. sativa* seed (tk, E), *N. sativa* seed (ir), *N. sativa* seed (T), *N. sativa* seed (M)	Tr, ND, ND, ND, 38.3	(Bourgou et al., 2010b; Jrah Harzallah et al. (2011); Moretti et al., 2004; (Piras et al., 2013)
β-Elemene	*N. arvensis* seed, *N. damascena* seed (M), *N. sativa* seed (M)	69.0, 73.2, 5.5	(Moretti et al., 2004; Edris, 2009)

ND, Not detected/reported; C, China; I, India; Ir, Iran; E, Egypt; M, Morocco; T, Tunisia; Tk, Turkey; Tr, traces.

^a^ Depending on the extraction method and conditions.

^b^ Depending on the origin.

The essential oil composition is highly variable and probably more chemotypes exist. As an example, Jrah Harzallah et al. (2011) identified eighty-four compounds in the essential oil obtained by hydrodistillation of *N. sativa* seeds from Tunisia and the major one was the monoterpenes *p*-cymene (9) (49.48%), α-thujene (15) (18.93%), α-pinene (16) (5.44%), β-pinene (16) (4.31%). Alternatively, the bioactive compound thymoquinone represented only 0.79%. Similar results were found by Geng et al. (2009); *N. sativa* seeds contained *p*-cymene (9) (33.75%) as the major component using the hydrodestillation mode, with low content of thymoquinone (8) (3.73%), while using supercritical CO_2_ extraction the major one was linoleic acid (1). Another study reported that *N. sativa* seeds from Tunisia and Morocco showed again that *p*-cymene (9) occurred in a higher relative concentration (60.5 and 56.7%, respectively) (Bourgou et al., 2010b; Badri et al., 2018). Furthermore, the essential oil extracted by hydrodistillation from Egyptian seeds showed the major components were *p*-cymene (9) (33.0%) and thymoquinone (8) (32.2%), followed by α-thujene (15) (13.0%) and camphene (19) (2.9%) (Viuda-Martos et al., 2011). Rarely, the composition of *N. sativa* seeds essential oil from Poland showed that two other monoterpenoids were present: *cis*- and *trans*-4-methoxythujane (18) (Wajs et al., 2008). Bourgou et al. (2012) has identified also four terpenoids, *trans/cis*-sabinene hydrate methyl ether (20), 1,2-epoxy-menth-4-ene (21), and 1,2-epoxy-menth-4(8)-ene (22), in Tunisian *N. sativa* essential oil from seeds by nuclear magnetic resonance (Figure 2).

*Nigella damascena* seed oil was characterized by almost 100% sesquiterpenes, of which β-elemene (17) (73.2%) was the most representative one (Moretti et al., 2004; Geng et al., 2009). Edris (2009) also remarked that this compound could be present even up to 73.0% in the essential oils of in *N. orientalis* and *N. arvensis*. Alternatively, *N. arvensis* could also contain other compounds in major levels, e.g. a methylated derivative of carvacrol (19) (26.4%), carvacrol methyl ether, followed by β-pinene (16) (21.4%).

### Alkaloids

**FIGURE 3:**
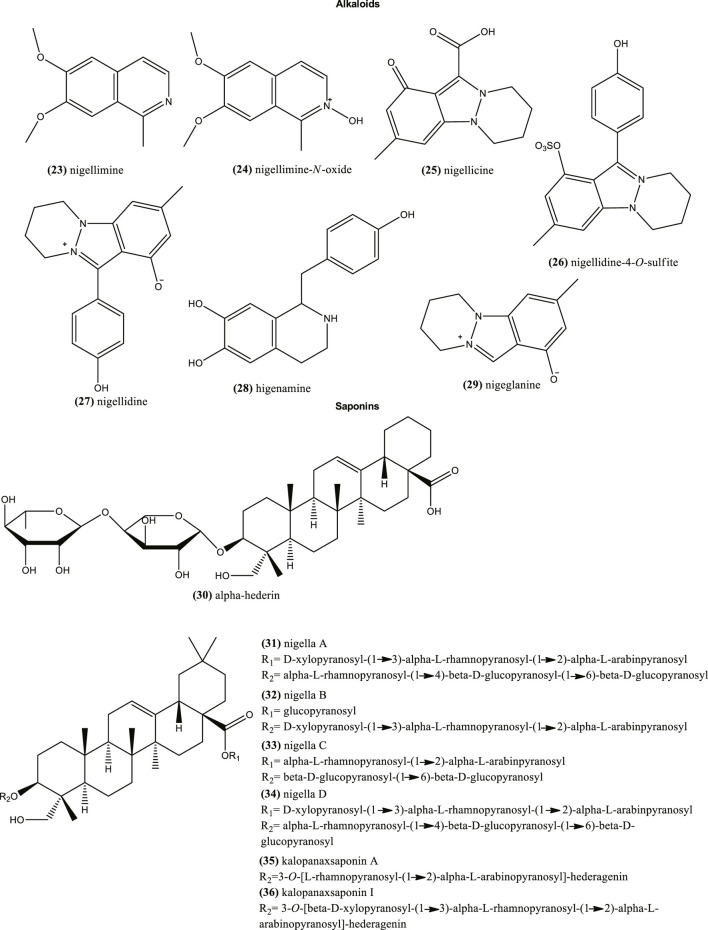
Example of chemical structures of alkaloids and saponins reported in *N. sativa*.

The bio-potential of nitrogen-containing heterocycles has been recently revised (Thakral and Singh, 2019). In plants, alkaloids contain one or more nitrogen atoms and usually are situated in some cyclic system. Similarly, these nitrogen-containing heterocycles, such as isoquinoline alkaloids and their *N*-oxides, can be a source of leads for drug discovery (Dembitskya et al., 2015). This included compound such as nigellimine (23) and nigellimine-*N*-oxide (24) from *N. sativa* seeds. This species also contains other type of alkaloids such as nigellicine (25), and nigellidine-4-*O*-sulfite (26) (Figure 3). Nigellidine (27) and its derivative methyl nigellidine, higenamine (28), and nigeglanine (29) were also characterized in *N. sativa* seeds (Atta Ur et al., 1995; Yun et al., 2014). Among them, methyl nigellidine, nigeglanoside and nigelloside could be used as markers to differentiate both species (Yun et al., 2014).

### Saponins

Other studies also suggest the presence of phenolic compounds and triterpenes like saponins in the seeds of *N. sativa* and other *Nigella* species (Ali et al., 2008; Atta Ur, 1985; Atta Ur, 1992; El-Naggar et al., 2017; Kooti et al., 2016; Tiruppur Venkatachallam et al., 2010; Zribi et al., 2014). Saponins are a heterogeneous group of glycosides, which have one or more hydrophilic moieties combined with a lipophilic triterpene or steroid derivative. In particular, triterpene saponins are highly characteristic compounds in *Nigella* seeds. Besides alpha-hederin (30) (Boubertakh et al., 2013), other relative saponins are: 3-*O*-[d-xylopyranosyl-(1→3)-α-l-rhamnopyranosyl-(1→2)-α-l-arabinpyranosyl]-28-*O*-[-α-l-rhamnopyranosyl-(1→4)-β-d-glucopyranosyl-(1→6)-β-d-glucopyranosyl] hederagenin (nigella A) (31), 3-*O*-[d-xylopyranosyl-(1→3)-α-l-rhamnopyranosyl-(1→2)-α-l-arabinpyranosyl]-28-*O*-β-d-glucopyranosyl hederagenin (nigella B) (32), 3-*O*-[α-l-rhamnopyranosyl-(1→2)-α-l-arabinpyranosyl]-28-*O*-[β-d-glucopyranosyl-(1→6)-β-d-glucopyranosyl] hederagenin (nigella C) (33), 3-*O*-[d-xylopyranosyl-(1→3)-α-l-rhamnopyranosyl-(1→2)-α-l-arabinpyranosyl]-28-*O*-[-α-l-rhamnopyranosyl-(1→4)-β-d-glucopyranosyl-(1→6)-β-d-glucopyranosyl] hederagenin (nigella D) 34) (Figure 3). The latter were reported in the seeds of *N. sativa* and exhibit a broad spectrum of bioactivities (Chen et al., 2018; Dönmez and Mutlu, 2004). Others also found in the latter species are: 3-*O*-[l-rhamnopyranosyl-(1→2)-l-arabinopyranosyl]-hederagenin (kalopanaxsaponin A) (35) and 3-*O*-[-d-xylopyranosyl-(1→3)-l-rhamnopyranosyl-(1→2)-l-arabinopyranosyl]-hederagenin (kalopanaxsaponin I) (36) (Figure 3), with anticancer properties reported *in vitro* (Tian et al., 2006).

### Phenolic Compounds

**FIGURE 4:**
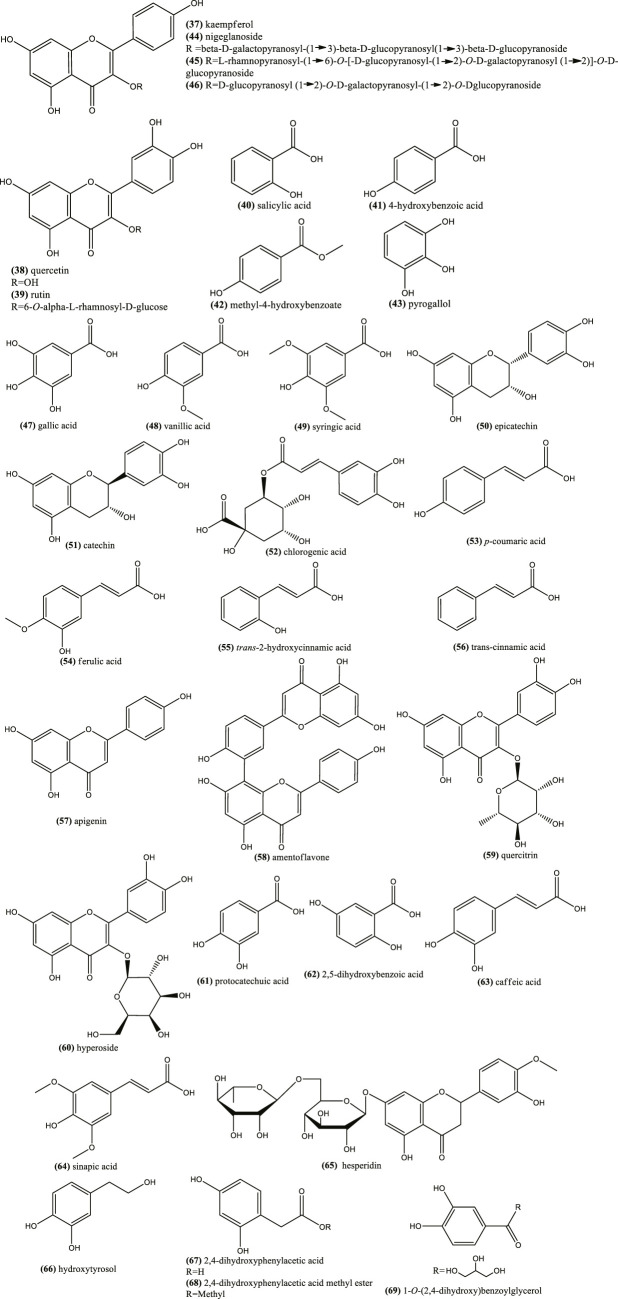
Example of chemical structures of phenolic compouds reported in *N. glandulifera*, *N. sativa*, *N. damascena,* and *N. arvensis*.

Concerning phenolic compounds, a recent study suggested that *N. sativa*, *N. arvensis*, *N. damascena*, and *N. hispanica* seeds have more flavonoids and phenolic acid derivatives than *N. nigellastrum* and *N. orientalis* seeds (Farag et al., 2014). Phenolic compounds are characterized to be small molecules with at least one phenol unit. Some common ones have been reported in the seeds of *N. sativa* seeds, e.g. kaempferol (37), quercetin (38), rutin (39), salicylic acid (40), *p*-hydroxybenzoic acid (41), methyl-4-hydroxybenzoate (42) and pyrogallol (43) (Xin et al., 2008; Boubertakh et al., 2013) (Figure 4). Table 3 shows examples of phenolic compounds described in *Nigella* plants and their contents.

**TABLE 3:** Example of phenolic compounds found in *Nigella* species.

Compound name	Source (origin)	Amount (mg/g)	References
*p*-Hydroxybenzoic acid	*N. sativa* seed (C)*, N. sativa* seed (T)	NR, 0.002	(Bourgou et al., 2008; Bourgou et al., 2010a)
Gallic acid	*N. sativa* seed (T), shoot (T), root (T)	0.3–1.0^a^, 0.3, 0.3	(Bourgou et al., 2008; Bourgou et al., 2010a)
Chlorogenic acid	*N. sativa* seed (T), shoot (T), root (T)	0.02–0.04^a^, 0.015, 0.004	(Bourgou et al., 2008; Bourgou et al., 2010a)
Syringic acid	*N. sativa* seed (T)	0.01–0.02^a^	(Bourgou et al., 2008; Bourgou et al., 2010a)
Vanillic acid	*N. sativa* seed (T), shoot (T), root (T)	2.2–3.5^a^, 1.4, 0.9	(Bourgou et al., 2008; Bourgou et al., 2010a)
*p*-Coumaric acid	*N. sativa* seed (T), root (T)	0.01–0.02^a^, 0.004	(Bourgou et al., 2008; Bourgou et al., 2010a)
Ferulic acid	*N. sativa* seed (T), root (T)	0.04–0.13^a^, 0.002	(Bourgou et al., 2008; Bourgou et al., 2010a)
*trans*-Cinnamic acid	*N. sativa* seed (T), shoot (T), root (T)	0.03–0.05^a^, 0.2, 0.01	(Bourgou et al., 2008; Bourgou et al., 2010a)
(–)-Epicatechin	*N. sativa* seed (T), shoot (T), root (T)	0.01–0.02^a^, 0.01, 0.01	(Bourgou et al., 2008; Bourgou et al., 2010a)
(+)-catechin	*N. sativa* seed (T)	0.1–0.3^a^	(Bourgou et al., 2008; Bourgou et al., 2010a)
Quercetin	*N. sativa* seed (C)*, N. sativa* seed (T), shoot (T), root (T)	NR, 0.002–0.005^a^, 0.03, 0.03	(Bourgou et al., 2008; Bourgou et al., 2010a)
Apigenin	*N. sativa* seed (T), shoot (T), root (T)	0.003–0.005^a^, 0.07, 0.02	(Bourgou et al., 2008; Bourgou et al., 2010a)
Amentoflavone	*N. sativa* seed (T), shoot (T)	0–0.001^a^, 0.03	(Bourgou et al., 2008; Bourgou et al., 2010a)
Quercetin	*N. damascena* seed (R), *N. damascena* seed (J), *N. arvensis* seed (J)	0.014, NR, NR	(Toma et al., 2015)
Quercitrin	*N. damascena* seed (R), *N. sativa* seed (R)	0.020, 0.004	(Toma et al., 2015)
Hyperoside	*N. damascena* seed (R)	0.001	(Toma et al., 2015)
Kaempferol	*N. arvensis* seed (J), *N. damascena* seed (J), *N. sativa* seed (C)*, N. sativa seed* (R)	0.006, NR, NR, NR	(Toma et al., 2015)
Kaempferol-3-*O*-[β-d-glucopyranosyl-(1ê2)-β-d-galactopyranosyl-(1ê2)-β-d-glucopyranoside]	*N. arvensis* seed (G), *N. damascena* seed (A, I), *N. sativa* seed (C), *N. hispanica* seed (G, F), *N. nigellastrum* seed (CR), *N. orientalis* seed (tk), *N. sativa* seed (E, et, S, SA, tk)	ND, tr, ND, ND, tr, 3.4–6.1^a^, NR	(Farag et al., 2014)

A, Austria; C, China; CR, Czech Republic; E, Egypt; Et, Ethiopia; F, France; G, Germany; I, Italy; J, Jordan; NR, Not reported; R, Romania; S, Syria; T, Tunisia; Tk, Turkey; SA, Saudi Arabia.

^a^ It depends on the salinity tested.

Kaempferol and quercetin derivatives are also common in other *Nigella* species (Farag et al., 2014). Moreover, characteristic glycosilated flavonoids, also found in the seeds, are: kaempferol 3-*O*-beta-d-galactopyranosyl-(1→3)-beta-d-glucopyranosyl(1→3)-beta-d-glucopyranoside (nigeglanoside) (44) (Hao et al., 1996), kaempferol 3-*O*-l-rhamnopyranosyl-(1→6)-*O*-[-d-glucopyranosyl (1→2)-*O*-d-galactopyranosyl (1→2)]-*O*-d-glucopyranoside (45) and kaempferol 3-*O*-d-glucopyranosyl-(1→2)-*O*-d-galactopyranosyl-(1→2)-*O*-d-glucopyranoside 46) (Figure 4), which were reported in *N. sativa* (Liu et al., 2011; Xin et al., 2008) (Figure 4). The latter flavonoid is a taxonomic marker for distinguishing *N. sativa* from other *Nigella* species (Farag et al., 2014).

Methanolic extracts from seeds, shoots and roots of Tunisian *N. sativa* contained phenolic compounds, including: gallic acid (47), *p*-hydroxybenzoic acid (41), vanillic acid (48), syringic acid (49), (–)-epicatechin (50), (+)-catechin (51), chlorogenic acid (52), *p*-coumaric acid (53), ferulic acid (54), *trans*-2-hydroxycinnamic acid (55), *trans*-cinnamic acid (56), quercetin (38), apigenin (57), and amentoflavone (58) (Figure 4). Nonetheless, among them, vanillic acid (48) was the major phenolic compound (Bourgou et al., 2008; Bourgou et al., 2010a).

A recent study has shown that there are qualitative and quantitative differences between the phenolic compounds of ethanolic extracts from *N. sativa* and *N. damascena* seeds (Toma et al., 2015). For example, quercitrin (59) was detected in both extracts, hyperoside (60) and quercetin (38) in *N. damascena*, while kaempferol (37) was found only in *N. sativa*. Moreover, a recent study has reported a more complex phenolic profile, which was found complexed with *N. damascena* seeds proteins: gallic acid (47), protocatechuic acid (61), 2,5-dihydroxybenzoic acid (62), vanillic acid (48), (+)-catechin (51), caffeic acid (63), chlorogenic acid (52), syringic acid (49), (–)-epicatechin (50), *p*-coumaric acid (53), sinapic acid (64), hesperidin (65), quercetin 38) and kaempferol (37).

Some of these compounds were also present in *N. arvensis* seeds (Alu'datt et al., 2016), while 7-methylkaempferol (rhamnocitrin) was also reported in the epigeal part of this species (Kirichenko et al., 1972). Other type of phenolic compounds reported in *N. damascena* seeds were hydroxytyrosol (66), 2,4-dihydroxyphenylacetic acid (67) and its methyl derivative (68), as well as a new phenolic ester, 1-*O*-(2,4-dihydroxy) benzoylglycerol 69) (Fico et al., 2000) (Figure 4).

### Others

**FIGURE 5:**
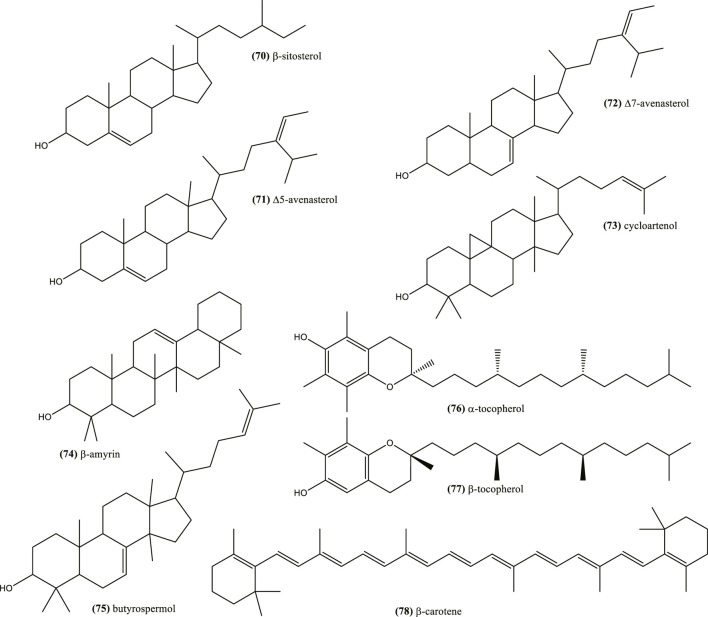
Example of phytosterols and stanols reported and other terpenes in *Nigella*.

Examples of other reported terpene chemicals include phytosterols such as β-sitosterol (70), ∆5-avenasterol (71), and ∆7-avenasterol (72), as well as stanols such as cycloartenol (73) (Figure 5). Other triterpenes like β-amyrin (74) and butyrospermol (75) (Ramadan and Mörsel, 2002; Ahmad et al., 2013; Ijaz et al., 2017), as well as α-tocopherol (76), γ-tocopherol (77), and β-carotene (78) are also found in seeds (Ramadan and Mörsel, 2004) (Figure 5).

### Factors that Affect the Phytochemical Composition

Phytoconstituents of *Nigella* may vary even within the same species and this is related to many factors such as growing and climatic conditions, location, different organs of the plants and the extraction methods used (Edris, 2010; Manju et al., 2016; Saxena et al., 2017) (Tables 2 and 3). Secondary metabolites, compounds which are not directly related to the development, growth and reproduction of plants but they have significant performance in chemical communication, primary defense against biotic and abiotic stress (Sarkar and Shetty, 2014) and also in epigenetic memory. The chemical composition may vary also during development stage and this was confirmed by Zribi et al. (2014) who demonstrated that the total phenolics, flavonoids, flavonols and flavones, alkaloids and proanthocyanidins contents of Tunisian and Indian *N. sativa* aqueous extracts were the highest in the vegetative stage. Moreover, the aerial parts from the two varieties were richer in total phenolics and flavonoids, including flavonols, flavones and proanthocyanidins, than seeds. Salinity is also another factor that greatly influenced the phenolic composition of *Nigella* seeds; e.g. generally the content of certain phenolic compounds decreased, including the major one vanillic acid (45), while the content of *trans*-cinnamic acid (53), quercetin (38), and apigenin (54) increased. This was related with a decrease of the antioxidant activity (Bourgou et al., 2010a). Today, this is essential information since water scarcity and salinization of arable lands may occur in a future scenario exposed to drastic changes caused by climate (Selim et al., 2019), affecting not only crop growth but also its phytochemical content and antioxidant properties.

This means that a high variability in the content of phytochemicals can be found in *Nigella* plant parts, and in particular in *N. sativa* seeds, and from a functional point of view each essential oil/extract should be further characterized in order to elucidate the real active molecules inside for further standardization and quality control.

## Biological Activities: Preclinical *In Vitro/In Vivo* Studies of the Genus *Nigella* and active compounds

This section collects the results of *in vitro, ex vivo,* and *in vivo* studies on the antioxidant, anticancer, cardioprotective, antidiabetic, antiobesity and neurological properties of *Nigella* species, mainly, *N. sativa*, as well as some active principles, including thymoquinone.

### Antioxidant Activity and Effects on Oxidative Stress

Some studies suggest that *N. sativa* oil and extracts have antioxidant properties and can modulate oxidative stress, which could be beneficial for oxidative stress related diseases/disorders. In this sense, oils and solvent extracts from *N. sativa* seeds possessed antioxidant activity *in vitro* (Toma et al., 2015; Mohammed et al., 2016; Abedi et al., 2017; Singhal et al., 2017), *ex vivo* (Ghoreyshi et al., 2020), *in vivo* (Mahmoudi et al., 2018; Rasoli et al., 2018) and in humans (Nikkhah-Bodaghi et al., 2019). Moreover, *in vivo* regular intake of ethanolic extract of *N. sativa* seed (400 mg/kg) lowered the lipid peroxidation, and enhanced catalase activity in Wistar rats (Rasoli et al., 2018). Another work suggested that hydroalcoholic and hexane extracts of *N. sativa* seed as well as thymoquinone may counteract oxidative stress caused by high-fat diets (HFDs), e.g. malondialdehyde (MDA) levels decreased, while the activity of catalase enzyme and serum total antioxidant capacity increased (Mahmoudi et al., 2018). Concerning other *Nigella* species, *N. damacena* 70% ethanolic extract (seeds) has shown antioxidant properties *in vitro*, even higher generally than *N. sativa* seeds (Toma et al., 2015); the former contained higher amounts of quercetin derivatives. Also, the fixed oil from the seeds of another species, *Nigella unguicularis* Spenner, showed favorable oxidant/antioxidant balance and blood lipids profile when it was administered to rats (1 ml/kg orally for 4 weeks) (Kökdil et al., 2005). However, the responsible active compounds were not determined in the latter case.

As commented before, thymoquinone can have a protective role against oxidative stress (Woo et al., 2013). Thymoquine has shown antioxidant properties *in vitro*, in particular using the oxygen radical absorbance capacity (Tesarova et al., 2011). Essential oil extracted from *N. sativa* seeds with higher thymoquinone content showed stronger antioxidant activity than other essential oils (Abedi et al., 2017). In this context, Usta and Dede (2017) assessed role of thymoquinone on oxidative DNA damage and NF-κB levels in diabetic rats. The results revealed that the oxidative DNA damage (8 hydroxy-2-deoxyguanosine) and NF-κB levels were insignificantly lowered after treatment. Moreover, it also reduced glycosylated hemoglobin, glucose levels and aspartate aminotransferase (AST), alanine aminotransferase and gamma-glutamyl transpeptidase activities. In recent *in vitro* and *in vivo* studies, thymoquinone in combination with iron oxide nanoparticles showed attenuation in genetic and oxidative damage, and caused an enhancement in the levels of anti-oxidant enzymes (Ansari et al., 2019).

Nonetheless, other *Nigella* phytochemicals constituents have antioxidant activity, which is not strictly dependent on the thymoquinone content (Bordoni et al., 2019). Thymohydroquinone (11) has also shown scavenging properties *in vitro*, while no effects were found for dithymoquinone (10) (Tesarova et al., 2011). *Trans*-anethole (14), isolated from the essential oil of *N. sativa* seeds by thin-layer chromatography, also showed free radical scavenging properties using several methods (Burits and Bucar, 2000). Moreover, the phenolic nature of thymol (13) and carvacrol (12) makes them to possess higher antioxidant activity than other volatile constituents in oils (see reviews by (Salehi et al., 2018; Sharifi-Rad et al., 2018; Salehi et al., 2019). In an antioxidant activity guided fractionation of the essential oil from *N. sativa* seeds it was found that thymoquinone 11) (51%), thymol (25%) and carvacrol (8%) were the main antioxidant compounds (Kazemi, 2015). Moreover, in oxidative stress induced experimental subjects, thymol (12) (thymol 10 mg/kg) has been shown significant impact on sperm quality via increasing the spermatozoa concentration and motility, and lowering the MDA level. It also decreases the dead sperm ratio and enhances the glutathione concentrations in the testicles, liver and kidney tissues (Fangfang et al., 2017). The antioxidative properties of thymol (12) and carvacrol (13), through improving the antioxidants, suppressing lipid peroxidation markers and ameliorating oxidative stress, have also been shown in other *in vivo* studies (Bakir et al., 2016; Samarghandian et al., 2016; Saravanan and Pari, 2016).

It should be notice that polar extracts from *Nigella* could have a wide spectrum of other phenolic compounds, which are antioxidant in nature (see section 4.5), and thus it should be taken into account in further studies. As an example, free and bound phenolic compounds, which were associated to *N. damascena* and *N. arvensis* proteins from the seeds, have shown antioxidant properties *in vitro* (Alu'datt et al., 2016). As noted before, *N. damascena* seeds contain quercetin derivatives, including quercetin aglycone, hyperoside and quercitrin, as well as the total phenolic content showed enhanced antioxidant properties (Toma et al., 2015). Furthermore, total saponins from *N. sativa* seeds increased the plasma superoxide dismutase (SOD) and glutathione peroxidase (GSH-Px) activities and decreased MDA level compared to control group in d-galactose-induced aging model (6–24 mg/kg). These seeds also showed (2,2-diphenyl-1-picrylhydrazyl) DPPH radical scavenging properties (Dönmez and Mutlu, 2004).

### Anti-cancer Properties

*Nigella sativa* has anti-proliferative, pro-apoptotic, anti-oxidant, cytotoxic, anti-mutagenic, and anti-metastatic effects, underlined by several mechanisms reviewed by (Majdalawieh and Fayyad, 2016). Studies in cell lines, including in human lung (A-549 cells), epithelial cervical (HeLa and SiHa) and Michigan Cancer Foundation-7 (MCF-7) breast cancer cells, have shown that the administration of *N. sativa* alcoholic seed extracts, seed oil and nanoemulsions markedly lowered the cell viability, inducing apoptotic cell death, and/or altered the cellular morphology, with IC_50_ values between 0.41-82 μL/mL in some cases (Shafi et al., 2009; Hasan et al., 2013; Al-Sheddi et al., 2014; Periasamy et al., 2016; Butt et al., 2019). Among these studies, Hasan et al. (2013) revealed the presence of thymoquinone as the potential active *Nigella* constituent. Concerning *in vivo* studies, supplementation of different doses of *N. sativa* ethanolic extract (seed) on daily basis (150, 250, 350 mg/kg), thymoquinone (20 mg/kg), and daily dose of silymarin (100 mg/kg) prevented from the hepatocellular carcinoma proliferation provoked by diethylnitrosamine through multiple pathways: reduction in *p*-EGFR and *p*-ERK1/2, deactivation of EGFR/ERK1/2 signaling, down regulation of target genes (c−fos, PCNA, and Bcl2), suppression of cell proliferation, reduction in alpha-fetoprotein (AFP) and hepatic enzymes, and enhancement in the levels of antioxidant enzymes (Shahin et al., 2018). Other *in vivo* studies suggested a protective effect of *N. sativa* seeds oil and thymoquinone against breast carcinogens (Linjawi et al., 2015) with dosages between 1 and 10 mg/kg. The water extract of *N. sativa* has shown immunomodulatory and anti-tumor effects on Ehrlich ascites carcinoma in mouse model (Aikemu et al., 2013), but its composition was not characterized.

**FIGURE 6:**
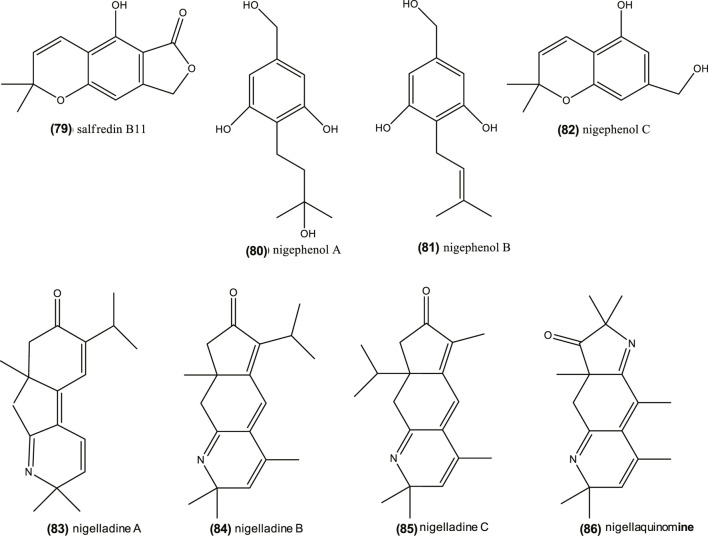
Chemical structure of salfredin B11, nigephenol A-C and of nigelladine A–C and nigellaquinomine.

Thymoquinone is one of the potential active compounds in *Nigella*, as commented before (Woo et al., 2012; Siveen et al., 2014; Shanmugam et al., 2018a; Shanmugam et al., 2018b). It also demonstrated anti-tumor activity on human liver HepG2 cancer cells lines (IC_50_ = 46 μM) by: enhanced caspase-3 enzyme activity, decreased MDA contents, induced cell apoptosis, and inhibited cell growth (Ismail et al., 2018). This compound also revealed to be a potential therapeutic adjuvant in human breast cancer cell lines (MCF-7, IC_50_ = 64.93 µM, and T47D, IC_50_ = 165 µM). In other cases, thymoquinone in combination with anti-cancer drugs, can exhibit antagonistic and synergistic effects (Bashmil et al., 2018). Nonetheless, as shown in “Phytoconstituents” section, *Nigella* genus is a rich source of phytochemicals, including dithymoquinone (10), thymohydroquinone (11), carvacrol (12), thymol (13), nigellimine-*N*-oxide (24), nigellicine (25), nigellidine (27), which may contribute to the anticancer properties (see reviews by Majdalawieh and Fayyad, 2016; Salehi et al., 2018; Sharifi-Rad et al., 2018; Salehi et al., 2019). Edris (2009) also remarked that β-elemene (17), which is one of the most abundant compounds in *Nigella* species like *N. orientalis*, *N. damascena,* and *N. arvensis*, has also anti-cancer properties. Moreover, recent studies suggest that *Nigella* saponins, including α-hederin (30), nigella A (31) and B (32), have an anti-cancer protective role (Chen et al., 2018; Dönmez and Mutlu, 2004; Kumara and Huat, 2001; Rooney and Ryan, 2005; Tian et al., 2006). Remarkably, nigella A (31) and B (32) (40 mg/kg, intragastric administration), extracted from *N. sativa,* inhibited tumor growth in nude mice by 42.82 and 37.20%, respectively, when compared with vehicle-administered animals (Chen et al., 2018). Furthermore, phenolic derivatives isolated in the seeds of *N. sativa*, salfredin B11 (79), nigephenol A (80) and B (81), and nigephenol C (82) (Figure 6), showed inhibition against HepG2 cells (Sun et al., 2015). Globally, seeds, their extracts and essential oils from *Nigella* are complex sources of anti-cancer compounds, whose level and synergism/antagonism require further study.

### Cardioprotective Properties

*Nigella sativa* has a high potential for the prevention and treatment of cardiovascular diseases, while little is known about the cardiovascular properties of other *Nigella* species. As an example, *N. sativa* seeds powder improved lipid profile and prevented atherosclerosis in hypercholesterolemic rabbits when it was supplied in their diet (5%). It significantly decreased fatty streak formation, total cholesterol and low-density lipoprotein (LDL) (Asgary et al., 2013). A recent study also suggests that diabetic rats treated with an ethanolic extract from *N. sativa* seeds (100–400 mg/kg) for six weeks reduced serum glucose and lipids, while it improved atherogenic index of plasma, vasoreactivity, endothelial dysfunction, and vascular inflammation (Abbasnezhad et al., 2019). *Nigella sativa* (seed, oil and extracts) has a potential role in the management of hypertension, as shown *ex vivo* (Hebi et al., 2016; Abbasnezhad et al., 2019) and *in vivo* studies (Khattab and Nagi, 2007; Leong et al., 2013; Enayatfard et al., 2018) due to vasorelaxant properties. It also may increase endothelial nitric oxide (NO) synthesis, attenuate cardiovascular effects of the vasoconstrictor angiotensin II, and inhibit the parasympathetic tone (Khattab and Nagi, 2007).

*Nigella sativa* seed oil used in the study by Asgary et al. (2013) contained predominantly terpenoids, including *p*-cymene 9) (37.3%) and thymoquinone 8) (13.7%). In other studies, *N. sativa* seeds extracts contained thymoquinone (8), which were standardized (0.05–0.06% extract weight) (Enayatfard et al., 2018; Abbasnezhad et al., 2019). In fact, thymoquinone has demonstrated very good cardioprotective benefits and antihypertensive effects (Enayatfard et al., 2018). Liu et al. (2019) reported that thymoquinone has protective effects against cardiac damage in BALB/c mice via multiple mechanisms including reduction in intestinal histological alterations, suppressions of p62, NLRP3, IL-1β, TNF-α, caspase-1, IL-6 and 18, as well as MCP-1 expressions, inhibition of troponin-T levels in serum, enhancement in ATP, improvement in IL-10 and beclin 1 levels, and decrement in phosphatidylinositide 3-kinase level. Convincingly, thymoquinone effectively modulates pyroptosis, autophagy, and pro-inflammatory markers in cardiac stress (Liu et al., 2019). Likewise, thymoquinone reverted doxorubicine-induced cardiotoxicity when administered to mice. of the administration of thymoquinone (10–20 mg/kg p.o) significantly ameliorated oxidative stress markers involving lipid peroxidation, creatine kinase (CK)-MB, lactate dehydrogenase (LDH), and AST. Alongside, meaningful improvement in antioxidant enzymes like SOD, catalase, glutathione reductase, and glutathione-S-transferase has also been noticed (Alam et al., 2018). Another recent study reported by Atta et al. (2018) showed the protective role of thymoquinone on diabetes-caused cardiac complications in Wistar male rats. Lu et al. (2018) reported that thymoquinone (8) provides shield against myocardial ischemia/reperfusion (I/R) injury in isolated rat hearts and neonatal rat cardiomyocytes models via: improving left ventricular function, lowering myocardial infarct size, attenuating mitochondrial oxidative damage, producing LDH, and lowering MDA levels and H_2_O_2_ concentrations, alongside improving antioxidant enzymes levels. Additionally, the cardioprotective role of this compound was also linked to the up-regulation of sirtuin 1 (SIRT1) expression and inhibition of p53 acetylation (Lu et al., 2018). Thymoquinone 8) showed prophylactic effect from myocardial I/R injury in Langendorff perfused rat hearts. It encouraged autophagy, favored cardiac function, lowered infarct size, LDH and CK-MB levels, and suppressed oxidative stress (Xiao et al., 2018). Finally, Salahshoor and their co-workers highlighted that the supplementation of thymoquinone (4.5–18 mg/kg) in mice significantly increased the mean diameter of central hepatic vein, blood serum NO level, and liver enzymes level, while it decreased the liver weight (Salahshoor et al., 2018).

Two other interesting molecules are again carvacrol (12) and thymol (13). The latter was a cardioprotective agent against carotid tissue of hypercholesterolemic rats. The supplementation of thymol (24 mg/kg) in experimental subjects prevents from cardio complications through several mechanisms such as reduction in low density lipoprotein, triglycerides, increment in high-density lipoprotein level, reduction in apoptotic proteins and inflammatory expressions, phosphorylation of p38 (p-p38) and the protein expression of cleaved caspase-3 (Bayatmakoo et al., 2017). In another work, thymol (13) administration (7.5 mg/kg) reversed changes produced by isoproterenol in rats. It also enhanced the caspase-8 and 9, as well as Fas genes expression while lowered Bcl-xL gene expression in myocardium (Meeran et al., 2016). Among other effects, previous investigation revealed that multiple pathways are involved in this effect: a lowered serum cardiac troponin-Y, lysosomal thiobarbituric acid reactive substances (TBARS), high sensitivity C-reactive protein (hsCRP), and lowered activities of β-glucuronidase, β-galactosidase, cathepsin-B and D activities in lysosomes. Thymol down-regulated the proinflammatory cytokines involving IL-6 and 1β genes expression and TNF-α in myocardium of rats, and lowered heart weight, left ventricular hypertrophy and increased ST segments and tachycardia (Nagoor Meeran et al., 2015a; Nagoor Meeran et al., 2015b; Nagoor Meeran et al., 2016). El-Sayed et al. (2016) evidenced the protective role of both thymol (13) (20 mg/kg p.o.) and/or carvacrol (12) (25 mg/kg) against doxorubicin induced cardiotoxicity, ameliorating the heart function and oxidative stress parameters.

### Antidiabetic

*Nigella sativa* could play a role against diabetes and obesity, and thereby it could be an interesting agent to treat/prevent the metabolic syndrome. In cell *in vitro* assays, the ethanol extract from *N. sativa* seeds has shown antidiabetic activity using adipocytes (Benhaddou-Andaloussi et al., 2008; Benhaddou-Andaloussi et al., 2010). In particular, it was able to activate the AMPK pathway and the insulin signaling pathway, as well as it acted as an agonist of the peroxisome proliferator-activated receptor (PPARγ) (Benhaddou-Andaloussi et al., 2010), which plays a role in the regulation of metabolism. The seed oil from *N. sativa*, which contained compounds such as thymoquinone (8), anethole (14), *p*-cymene (9), saturated and unsaturated fatty acids, had also antidiabetic properties *in vivo*. In this concern, *N. sativa* oil (500 mg/kg/day) was co-administered orally along with high-fructose water (20%, w/v) for 45 days. It was able to reduce fructose-induced insulin resistance by reduction of hepatic insulin-degrading enzyme protein and activation of insulin receptor signaling. It also decreased body weight (BW), serum lipids, and glucagon (Elseweidy et al., 2018). In addition, *n*-hexane and petroleum ether from the seeds of *N. sativa* has shown inhibitory properties against protein tyrosine phosphatase 1B, with half maximal inhibitory concentration values of 33.15 and 18.50 μg/mL, respectively. This could be another mechanism of action since this enzyme is involved in the down regulation of insulin and leptin signaling and thus this plant could have antidiabetic potential (Xin et al., 2010).

Among the bioactive constituents of *Nigella*, the antidiabetic and antiobesity properties of thymoquinone have been also revealed. The administration (20 mg/kg/day) to diet-induced obesity mice was able to reduce decrease fasting blood glucose (FBG) and insulin levels, and enhanced glucose tolerance and insulin sensitivity. Moreover, this compound decreased the level of liver triglycerides and serum cholesterol, increased protein expression of phosphorylated Akt, decreased serum levels of inflammatory markers, and decreased NADH/NAD^+^ ratio. It was related to the capacity of thymoquinone to increase insulin sensitivity in insulin-resistant HepG2 cells through a SIRT1-dependent mechanism (Karandrea et al., 2017). Glycemic parameters were momentously controlled by the administration of thymoquinone (8) to the diabetic rats (Usta and Dede, 2017). El-Aarag et al. (2017) also investigated the effects of this compound to boost anti-diabetic properties of metformin in streptozotocin-induced diabetes in male rats. Their investigation unveiled that negative impacts of streptozotocin were corrected and normal biochemical functions were restored by combined treatment of thymoquinone (8) and metformin. This combination also up-regulated the expression level of glucose transporter-2 (Glut-2). The elevated MDA level was reduced in liver homogenates of the thymoquinone (8) treated rats (Zribi et al., 2014). Thymoquinone (8) in nano-formulation (20–80 mg/kg) showed significant decrease in blood glucose and HbA1c levels and showed a dose dependent antihyperglycemic effect. It can result in better antihyperglycemic effect in type-2 diabetic rats than the non-capsulated compound (Rani et al., 2018). In another research, intraperitoneal administration of thymoquinone (8) (50 mg/kg) resulted in amelioration of dyslipidemia, hypoinsulinemia, hyperglycemia, impaired antioxidant defense system and upregulation of the expression of PPAR-γ and GLUT4 genes in diabetic rats (Moneim et al., 2018).

Concerning thymol (13), intragastric administration (40 mg/kg/day) for the subsequent 5 weeks momentously lowered kidney weight and blood and urinary markers of kidney injury by HFD-induced nephropathy in diabetic mice (Saravanan and Pari, 2016). It further reduced the HbA1c, leptin and adiponectin levels in the mice receiving thymol treatment. The plasma triglyceride, cholesterol, LDL and free fatty acids were reported to be significantly lowered and HDL was momentously elevated in thymol treated mice (Saravanan and Pari, 2016). It also represented beneficial effects on HFD-induced cognitive deficits via activating Nrf2/HO-1 signaling and improving hippocampal insulin resistance (Fangfang et al., 2017). The neuroprotective effect of carvacrol (12) has been also demonstrated on diabetes-associated cognitive deficit in a rat model of diabetes treated with this compound (25–100 mg/kg, 7 weeks). It prevented behavioral, biochemical, and molecular changes associated with diabetes in a dose-dependently way (Deng et al., 2013).

However, the aforementioned *in vitro* study on adipocytes (Benhaddou-Andaloussi et al., 2010) suggests that compounds other than thymol, such as carvacrol (12), hederin (30), nigellimine (23), and thymoquinone (8), could be active forms. In this regards, four alkaloids, nigelladines A–C (83–85) and nigellaquinomine 86) (Figure 6) have been isolated in *N. sativa* seeds and possessed inhibitory activity protein tyrosine phosphatase 1B (PTP1B) inhibitory activity, which is key negative regulator of insulin signaling (Chen et al., 2014). Active *n*-hexane and petroleum ether extracts from this plant were also rich in palmitic acid and unsaturated fatty acids like oleic and linoleic acids, and their methyl esters (Xin et al., 2010).

### Antiobesity

*Nigella sativa* fruit and seed extracts and oil (33% thymoquinone) have shown antiobesity potential using *in vitro* and *in vivo* studies, and the mechanisms include the inhibition of pancreatic lipase and α-amylase (15–100%, depending on the extract and enzyme), proinflammatory cytokine production in pre-adipocytes (a model of low-grade inflammation in Simpson–Golabi–Behmel syndrome human) and weight loss, by positively affecting the uncoupling protein-1 (UCP-1), which the index protein of the brown adipose tissue used in the obesity studies (Buchholz and Melzig, 2016; Mahmoudi et al., 2018; Bordoni et al., 2019).

The latter *in vivo* study, which was performed in mice feed with HFD, suggested that the weight loss effect was less dependent on tymoquinone, while other phenolic compounds may affect (Mahmoudi et al., 2018). Alternatively, studies on female C57BL/6 mice, which were also subjected to HFD and supplemented with thymoquinone (10–20%), showed that this compound ameliorated obesity-induced metabolic dysfunction and impaired positive effects on ovarian and mammary gland metabolic functions (Harphoush et al., 2019). In another context, obesity markers were significantly down-regulated while momentous control over obesity-induced changes in biochemical profile of the body by thymol (13) treatment. It also modified fat and glucose metabolism in such a way to favor the control over obesity in mice partly via hypolipidemic, hypoglycemic, hypo-insulinemic, hypoleptinemic, and pancreatic lipase inhibition action (Haque and Ansari, 2018). This compound has also been endorsed to possess anti-obesity properties by controlling obesity markers in HFD induced obese male Wistar rats. They recorded significant reduction in body and liver weights, visceral fat pad weight, food intake, leptin and inhibition of pancreatic lipase (Haque et al., 2014). Among molecular mechanisms, Choi et al. (2017) assessed the role of thymol (13) in 3T3-L1 white adipocytes via promoting mitochondrial biogenesis, enhancing expression of a core set of brown fat-specific markers as well as enhancing the protein levels of PPARγ, PPARδ, pAMPK, pACC, HSL, PLIN, CPT1, ACO, PGC-1α, and UCP1. It also augmented lipolysis, thermogenesis and fat oxidation. In the case of carvacrol (12), it can act by reduction of autophagy (essential for adipocyte maturation) and on carbohydrate-responsive element-binding protein ChREBP activity, reducing adipogenic differentiation (Spalletta et al., 2018). The supplementation of this compound in the diet (0.1%) may prevent diet-induced obesity by modulating gene expressions involved and protein associated with the signaling cascades in adipogenesis, as well as inflammation (Cho et al., 2012). Overall, it seems that *N. sativa* has an interesting profile of phytochemicals, which can act in different ways and require attention.

### Neuroprotection

The role of *N. sativa* and its components as promising neuropharmacological agents (Beheshti et al., 2016) in facitilating learning and memory have been recently reviewed (Sahak et al., 2016). In rats, 1 ml/kg of *N. sativa* oil for 14 days depleted reactive oxygen species (ROS)/NO levels, improved neurogenic proteins, acetylcholinesterase (AChE) activities and neuro-cognitive markers depletions in chlopyrifos exposure (Imam et al., 2018). Moreover, *N. sativa* oil improved neurocognicives indices such as Morris water maze. In other study performed in rats, the hydroalcoholic extracts of *N. sativa* seeds (200 and 400 mg/kg, 5 days) were used to investigate its effects on memory and brain tissues oxidative damage in penthylenetetrazole-induced repeated seizures (Vafaee et al., 2015). The results showed a beneficial effects of *N. sativa* on learning and memory impairments (Morris water maze) and improved antioxidant effects in the rat brain (lower MDA levels).

## Health-Promoting Effects: Clinical Trials in Humans

Clinical studies have mostly confirmed some effects of the seeds of *N. sativa* and their derivatives (seed extract and seed oil) obtained in the aforementioned *in vitro* and *in vivo* animal studies. Particularly, in the on line database about clinical trials “www.clinicaltrials.gov”, there are 16 studies on *N. sativa*. Among them, the status of four studies on blood lipids, obesity and diabetes management has been completed, while other two were about palmer arsenical keratosis and asthma. The administration of *N. sativa* seeds (or seeds oil/extract) was alone or with other herbal substances (e.g., *Curcuma longa* extract), with different dosages and up to 60 days. Some of the results were positive, e.g. in total cholesterol and asthma, and subject of publications (Qidwai et al., 2009; Koshak et al., 2017).

Furthermore, in the following section the results of other clinical studies regarding preventive and relieving effects of *N. sativa* on metabolic disorders and risk factors (diabetes, obesity and hypertension), as well as other effects are shown. In Table 4, we have summarized these effects. The effects of other *Nigella* plants are also commented.

**TABLE 4:** Clinical trials on effect of *Nigella sativa* to various system disorders, diseases and conditions.

Intervention	Application, duration	Type of study	Number of patients/study design^a^	Control	Main effects^a^	References	
Powdered *N. sativa* seed	Oral application for 8 weeks, 2 g of powder per day	Double-blind placebo controlled randomized clinical trial	40 patients with HT (aged between 22 and 50 years)	Placebo (starch)	↓ body weight and BMI, ↓ TSH and anti-TPO antibodies, ↑ T3, ↓ serum VEGF concentration	(Farhangi et al., 2016)	
*N. sativa* powder	Oral application for eight weeks	Randomized double-blind trial	40 patients with HT, 22–50 years	Placebo (starch)	↓ serum IL-23, ↓ TSH and anti-TPO antibodies, ↑ serum T3, ↓ body weight	(Tajmiri et al., 2016)	
*N. sativa* seed powder	Oral application for 8 weeks, 2 g per day	Double-blind placebo controlled randomized clinical trial	40 patients with HT (aged between 22 and 50 years)	Placebo (starch)	↓ serum LDL and T3, ↑ HDL	(Farhangi et al., 2018)	
*N. sativa* powdered seed	Oral application of 500 mg in combination with 500 mg of metformin, 10 mg atorvastatine, 150 mg aspirin	Randomized clinical trial	80 patients with metabolic syndrome and poor glycemic control (HbA1C>7%)	500 mg of metformin, 10 mg atorvastatine, 150 mg aspirin	↓ FBG, PPBG, HbA1c, LDL	(Najmi et al., 2012)	
*N. sativa* seed oil	Oral application for 3 months, 2.5 mL two times daily	Double-blind placebo controlled randomized clinical trial	70 patients with type II diabetes	Mineral oil	↓ FBG, PPBG, HbA1c	(Hosseini et al., 2013)	
*N. sativa* seed powder	Oral application of 2g powder daily, for one year in addition to their standard medications	Double-blind placebo controlled randomized clinical trial	114 patients with type 2 diabetes on standard oral hypoglycemic drugs	Placebo (charcoal)	↓ FBG, HbA1c, TBARS ↑ TAC, SOD, GSH	(Kaatabi et al., 2015)	
*N. sativa* oil soft gel capsules	Oral application of 3g oil daily, for 12 weeks	Double-blind placebo controlled randomized clinical trial	72 patients with diabetes type 2	Sunflower oil gel capsules	↓ FBG, HbA1c, TG, LDL	(Heshmati et al., 2015)	
*N. sativa* seed oil	Oral application of 2.5 mL two times daily for two months	Double-blind placebo controlled randomized clinical trial	68 healthy men 20–45 years of age with infertility lasting more than one year	Liquid paraffin	Sperm count, motility, morphology and semen volume, pH and round cells were improved significantly	(Kolahdooz et al., 2014)	
*N. sativa* seed extract	Two test groups received 100 and 200 mg of extract twice a day for 8 weeks	Double-blind placebo controlled randomized clinical trial	119 healthy male volunteers, aged 35 to 50	Placebo	↓ systolic and diastolic BP in a dose-dependent manner	(Dehkordi and Kamkhah, 2008)	
*N. sativa* seed oil	Oral application of 2.5 mL oil two times per day for 8 weeks	Randomized, double-blind placebo-controlled trial	70 healthy volunteers aged 34–63 years	Mineral oil	↓ systolic and diastolic BPs	(Fallah Huseini et al., 2013)	
*N. sativa* oil	6 mg/kg daily, for 30 days	Prospective and double-blind clinical study	66 patients with allergic rhinitis	Placebo	↓ nasal mucosal congestion, nasal itching, sneezing attack, runny nose, turbinate hypertrophy, and mucosal pallor during the first 2 weeks of the study	(Nikakhlagh et al., 2011)	
*N. sativa* nasal spray	2 puffs/day of *N. sativa* nasal spray (1 g/day of *N. sativa*) for 8 weeks	Randomized double-blind placebo-controlled trial	65 patients with mild to moderate chronic rhinosinusitis	Placebo (2 puffs/day of sodium chloride spray 0.65%)	Lund–McKay, lund Kennedy, and Sino-nasal outcome Test-22 scores significantly decreased in the intervention group	(Rezaeian and Amoushahi Khouzani, 2018)	
*N. sativa* oil	Topical application, twice a day for 6 months	Randomized, double-blind clinical trial	52 patients with vitiligo lesions	Fish oil	Reduction in size of lesions		(Ghorbanibirgani et al., 2014)
*N. sativa* ointment (2%)	Ointment (1 G) topically applied on eczematous lesions twice a day for a period of 4 weeks	Randomized double-blind placebo-controlled trial	60 patients with hand eczema, 18–60 years	Betamethasone and eucerin	↓ Dermatology life quality index score in *Nigella* and betamethasone groups compared to eucerin	(Yousefi et al., 2013)	
*N. sativa* seed powder and ointment	12 weeks, group I: 10% w/w ointment with *N. sativa* oil extract; group II: capsules with 500 mg of *N. sativa* powder, three times daily; group III: Combination of ointment and capsules	Randomized clinical trial	60 patients with mild to moderate plaque and palmoplanter psoriasis	-	Group I—total healing of psoriatic lesions, with good response in 65% of patients, and a relapse rate of 31% four weeks after cessation of treatment; group II—good response in 50% of patients, with a relapse rate of 50% observed four weeks after application; group III—total cure of lesions, and good responses in 85% of patients, with a relapse rate of 18%	(Jawad et al., 2014)	
*N. sativa* oil	Two placebo capsules daily for 1 month, followed by a month of NS oil capsules 500 mg twice per day	Placebo controlled clinical trial	40 female atients with rheumatoid artritis	Placebo (two starch capsules per day)	↓ disease activity score, ↓ number of swollen joints and the duration of morning stiffness	(Gheita and Kenawy, 2012)	
*N. sativa* oil	500 mg oil capsules two times daily for 8 weeks	Randomized, double-blind, placebo-controlled clinical trial	42 patients with rheumatoid artritis	Placebo	↑ IL-10, ↓ MDA, ↓ NO	(Hadi et al., 2016)	
*N. sativa* oil	Topical application twice a day (in the morning and night) for 21 days	Double-blind, parallel, clinical trial	52 pateints with osteoarthritis, 60–80 years	Diclofenac gel	Better pain relief effect compared to diclofenac gel according to KOOS score (38.88 ± 17.84 and 50.33 ± 20.38, respectively)	(Azizi et al., 2019)	
*N. sativa* seed	Oral administration: 500 mg in capsules (twice a day for 9 weeks)	Randomized study	20 healthy humans	Placebo: Psyllium seed husk in capsuels	Improvement in the parameters studied: Score of logical memory tests, attention test (letter cancenlation test and trail making test), cognitive test (Scroop)etc.	(Bin Sayeed et al., 2013)	

^a^BMI, body mass index; BP, blood pressure; FBG, fasting blood glucose; HbA1c, hemoglobin A1c; HT, Hashimoto’s thyroiditis; IL, interleukin; LDL, low density lipoprotein; MDA, malondialdehyde; PPBG, postprandial blood glucose; T3, total triiodothyronine; TAC, total antioxidant capacity; TBARS, thiobarbituric acid reactive substances; TG, triglycerides; TPO, thyroid peroxidase; TSH, thyroid-stimulating hormone; VEGF, vascular endothelial growth factor.

### Effect of N. sativa Supplementation on Patients with Metabolic Disorders and Risk Factors

#### Metabolic Syndrome

The possibility of application of *N. sativa* seed in patients with metabolic syndrome was firstly investigated by Najmi et al. (2008). They found that the administration with *N. sativa* seed oil (2.5 mL twice a day for 6 weeks) in patients with metabolic syndrome significantly decreased FBG and LDL and increased HDL levels. The same group of authors (Najmi et al., 2012) analyzed the effect of supplementation with powdered *N. sativa* seed (500 mg/day for two months) in patients with low glycemic control (glycate hemoglobin—HbA1C was lower than 7%). As a result, in the intervention group significant lowering of FBG, postprandial blood glucose (PPBG) and HbA1c was observed.

Conversely, a recent cross-over study (2 months treatment, 2 weeks washout period) evaluated the effects of *N. sativa*, as a form of traditional bread spiked with seeds (2 g), on FBG, BP and anthropometric indices (BW, waist circumference or WC, and BMI body mass index) in patients with metabolic syndrome. It found no significant effect between the mean of changes of parameters in the beginning and end of study (time effect), with the exception of diastolic BP (Mohtashami, 2019).

#### Diabetes

Oxidation and inflammation are important factors connected with occurrence of various chronic diseases, such as diabetes mellitus type 2 (Pouvreau et al., 2018). Due to the antioxidant, antiobesity and antidiabetic effects of *N. sativa* seeds and their constituents, this plant species can offer potential in prevention and treatment of type 2 diabetes. A recent review suggests that *N. sativa* can improve glycemic stages and lipid profile in diabetes (Heshmati and Namazi, 2015).

Hosseini et al. (2013) investigated anti-hyperglycemic effect of *N. sativa* seed oil in type II diabetic patients. As a result of supplementation with 5 mL of oil/day for three months, blood levels of fasting and 2 h PPBG, as well as HbA1c were significantly decreased compared to placebo group. This hypoglycemic effect can be result of an insulin senzitation and stimulation of pancreatic beta-cell function, resulting in intensified activity and consequent decrease in glucose level. Moreover, Kaatabi et al. (2015) reported pronounced antidiabetic activity after three-month application of *N. sativa* powdered seed (2 g/day) in combination with oral hypoglycemic agent in patients with type 2 diabetes. In this study, *N. sativa* received group showed significant reduction of FBG, HbA1C, and TBARS, and at the same time noticeable increase of the total antioxidant capacity, SOD, and glutathione levels were recorded in the intervention group. Heshmati et al. (2015) investigated effect of *N. sativa* seed oil application (3 g/day) on glucose metabolism and lipid concentrations in patients with type 2 diabetes. According to this study, FBG, HbA1C, and levels of triglycerides (TG) and LDL significantly changed in the intervention group compared to the placebo one. On the other hand, insulin level and its resistance decreased and HDL increased in the intervention group, but after adjusting for confounder factors, these parameters were not significant. Overall, the potential antidiabetic mechanisms of *N. sativa* could be mediated through a change in the oxidative status (either via upregulation of endogenous antioxidants or reduction of oxidative species), reduction of inflammation, and improvement of lipid profiles (Yimer et al., 2019). Furthermore, a recent meta-analysis confirms that supplementation with *N. sativa* could be a suitable choice to manage the complications of type 2 diabetes, including FBS (−17.84 mg/dL), HbA1c (−0.71%), total cholesterol (−22.99 mg/dL), and LDL (−22.38 mg/dL) (Daryabeygi-Khotbehsara et al., 2017).

#### Hypercholesterolemia and Obesity

*Nigella sativa* powder supplementation (1 g/day) for sixty days caused momentous reductions in concentrations of LDL, TG levels and enhancement in HDL level in hypercholesterolemic patients (Tasawar et al., 2011). Moreover, a double-blind, randomized, placebo-controlled 4-weeks trial showed that the administration of *N. sativa* seeds (1g/day) can reduce total cholesterol and LDL. Thus, it can be interesting as lowering lipid agent in hyperlipidaemic subjects (Pelegrin et al., 2019). *Nigella sativa* oil (3 g/day for 8 weeks) was also tested in a low-calorie diet on cardiometabolic risk factors in obese women. Compared to the placebo group, in the *N. sativa* treated group, weight (−6.0%) and WC (−6.9%) decreased. It also favored a reduction in triglyceride and LDL levels (Mahdavi et al., 2015). Recently, a recent meta-analysis (literature till June 2017) has been performed on the effects of supplementation with *N. sativa* on some anthropometric indices in adult subjects. It indicated that *N. sativa* supplementation exerts a moderate effect on reduction in BW (−2.11 kg), BMI (−1.16 kg/m^2^) and WC (−3.52 cm) (Namazi et al., 2018). Nonetheless, other meta-analysis performed till January 2018 suggested that *N. sativa* supplementation have only an effect on BW (−1.76 kg) and BMI (−0.85 kg/m^2^) in adults compared to placebo (Mousavi et al., 2018).

#### Hypertension

There are several clinical studies reporting positive effect of *N. sativa* application on hypertension. Dehkordi and Kamkhah (2008) demonstrated that application of *N. sativa* seeds extract (100 and 200 mg for eight weeks) in patients with mild hypertension led to significant reduction of systolic and diastolic BP compared to placebo. At the same time, a significant decrease in total and LDL cholesterol was observed and no other complications caused by the treatment were found. Similar results were obtained for *N. sativa* seed oil. Fallah Huseini et al. (2013) exhibited that its application in a dose 2.5 mL two times per day for 8 weeks reduced systolic and dyastolic BP in healthy volunteers. Authors suggested that the exhibited effect could be attributed to the activity of thymoquinone (8), one of main constituents of the volatile oil from *N. sativa* seed, as commented before.

Some of the cardiovascular benefits evidenced *in vitro* and now *in vivo* through risk factors, such as blood lipids, and BP, could be related to the presence of omega-6 fats as linoleic acid, which is the major fatty acid of the seed oil. Nonetheless, the cardiovascular health benefits of linoleic acid are controversial (Hooper et al., 2018; Marklund et al., 2019), and the positive effects of minor phytochemicals remains unclear.

### Other Effects of *N. sativa*

#### Effect of *N. sativa* Supplementation in Patients with Hashimoto’s Thyroiditis (HT)

HT is one of the most common human autoimmune diseases influencing the thyroid glands and an organ-specific T-cell mediated disease (Chistiakov, 2005). The disease is ten times more frequent in women than in men and it affects 2% of general population. HT is associated with serious alterations in composition and the transport of lipoproteins. Several clinical studies have demonstrated the efficacy of *N. sativa* application in patients with HT. According to the study conducted by Farhangi et al. (2016), the application of powdered *N. sativa* seeds (2 g) for eight weeks significantly decreased BW and BMI compared to placebo. Also, a positive effect has been achieved on thyroid function. A decrease in the serum concentrations of thyroid stimulating hormone (TSH) and anti-thyroid peroxidase (anti-TPO) has been shown, while serum concentrations of T3 increased in *N. sativa* treated group. In the same study, significant reduction of vascular endothelial growth factor (VEGF) was noted in the intervention group. It has been observed that its concentration grows during pathological states characterized by increased TSH secretion (Farhangi et al., 2016).

These results were confirmed by Tajmiri et al. (2016) and Farhangi et al. (2018), who showed lower BMIs in the group of patients with HT after the treatment with *N. sativa* seeds. This positive effect was exhibited through hypolipidemic effect: lowering serum LDL cholesterol and TG concentrations, while raising the level of HDL cholesterol. Beside this effect, Tajmiri et al. (2016) showed that treatment with *N. sativa* seed powder for eight weeks reduced levels of serum TSH, anti-TPO and IL-23, while level of serum T3 was increased.

#### Effect of *N. sativa* Supplementation on Patients with Reproductive System Disorders

Kolahdooz et al. (2014) studied effect of *N. sativa* seed oil on abnormal semen quality in infertile man. These authors showed that the daily application of 5 mL for two months significantly improved sperm count, morphology and motility and volume of semen, pH, and number of round cells. The exact mechanism of the exhibited effect is not determined, but it is likely related to strong antioxidant activity of the oil. Namely, it is well known that high level of oxidative stress contributes to decreased semen quality (Schulte et al., 2010).

#### Effect of *N. sativa* Supplementation on Patients with Allergic Rhinitis

The potential of application of *N. sativa* seeds and its oil in patients with allergic rhinitis has been also confirmed in clinical studies. Nikakhlagh et al. (2011) showed in prospective and double blind clinical trial that the oral administration of *N. sativa* seed oil (6 mg/kg daily) for 30 days in patients with allergic rhinitis significanly decreased the severity of respiratory symptoms, such as the presence of the nasal mucosal congestion, nasal itching, runny nose, sneezing attacks, turbinate hypertrophy, and mucosal pallor. Moreover, Rezaeian and Amoushahi Khouzani (2018) investigated the effect of *N. sativa* nasal spray in randomized clinical study. In this study patients in the intervention group received 2 puffs/day of *N. sativa* nasal spray (i.e. 1 g/day of *N. sativa*) and in the placebo group received 2 puffs/day of sodium chloride spray (0.65%). Lund–McKay, Lund Kennedy, and Sino-Nasal Outcome Test-22 scores were used and the outcomes were significantly lower in the intervention group compared to the placebo group. These findings are in line with previous reports about the antihistamine properties of *N. sativa* (Boskabady and Sheiravi, 2002; Kanter et al., 2006).

#### Effect of *N. sativa* Supplementation on Patients with Skin Diseases

Several clinical trials demonstrated positive effects of *N. sativa* application in resolving symptoms related to some skin diseases. Ghorbanibirgani et al. (2014) showed positive effects of 6 months topical application of *N. sativa* oil on vitiligo lesions compared to fish oil according to Vitiligo Area Scoring Index. In another study, Yousefi et al. (2013) investigated the therapeutic potential of 2% ointment topically applied (twice/day during 4 weeks) in patients with hand eczema compared to eucerin and betamethasone. According to scores obtained using Hand Eczema Severity index and Dermatology Life Quality Index, they showed that *N. sativa* ointment could have the same efficacy as betamethasone, while it is more efficient compared to eucerin. Jawad et al. (2014) investigated effect of various preparations of *N. sativa* in psoriasis treatment. A first group has been receiving *Nigella* ointment topically (10% w/w, twice daily), the second group took crude pulverized plant (capsules with 500 mg three times daily), while the third group received the combination of both treatments. The inspected reaction has been estimated following the Psoriasis Area and Severity Index score, while MDA serum level was used as indicator of the oxidative stress. After 12 weeks of treatment ointment reached total healing of psoriatic lesions, with good response in 65% of patients, and a relapse rate of 31% four weeks after cessation of treatment. Oral doses of *N. sativa* developed good response in 50% of patients, with a relapse rate of 50% observed four weeks after application. The combination of ointment and oral doses gained best results—total cure of lesions, and good responses in 85% of patients, with a relapse rate of 18%. All of these positive effects of *N. sativa* in dermatological diseases could be attributed to its antimicrobial, antioxidant, immunomodulatory, and anti-inflammatory effects.

#### Effect of *Nigella sativa* Supplementation in Patients with Rheumatic Artritis

The efficacy of black cumin oil in patients with rheumatoid arthritis (RA) was evaluated in female patients diagnosed with RA. Application of *N. sativa* oil in capsules (500 mg) twice daily exhibited improvement in disease activity score compared to placebo. Respectively, a pronounced improvement was demonstrated in amount of inflamed joints and occurrence of morning stiffness (Gheita and Kenawy, 2012). These results were confirmed by Hadi et al. (2016) with higher doses of *N. sativa* oil (1 g of oil/day, divided in two capsules). They showed that after 8 weeks of application in patients with RA noticeable decrease of serum MDA and NO was observed in the intervention group, while at the same time the serum level of anti-inflammatory cytokine IL-10 was increased (Hadi et al., 2016).

Azizi et al. (2019) compared the effects of topical application of *N. sativa* oil and diclofenac gel in patients with osteoartritis, one of the most common diseases in aging population. In this study, pain score has been expressed as average number obtained using Knee injury and Osteoarthritis Outcome Score scale. It has been observed that first ten days there has been no significant differences in placebo and the intervention group. Still, after 21 days of application better pain relief effect was observed after treatment with *N. sativa* oil compared to diclofenac gel.

#### Effects on Memory, Attention and Cognition

In a randomized study with 20 healthy humans, the effects of 500 mg of *N. sativa* seeds (twice a day for 9 weeks) on memory, attention, and cognition were studied. The results showed an improvement in the score of the tests studied, without an alteration of the biochemical markers of cardiac, liver and kidney functions (Bin Sayeed et al., 2013).

### Effects of Other *Nigella* Species

Among other herbs, there are several studies on the clinical use of *N. ciliaris* during childbirth and postpartum (Ali-Shtayeh et al., 2015), against diabetes (Ali-Shtayeh et al., 2012) and hypertension (Ali-Shtayeh et al., 2013), as well as *N. arvensis* against psoriasis (Jaradat et al., 2016b) and cancer (Jaradat et al., 2016a), which were performed in Palestine using questionnaires. Some patients seem to be satisfied with the outcomes of herbal therapy (Ali-Shtayeh et al., 2012; Ali-Shtayeh et al., 2013), but the authors did not reveal the herb treatment that was successful to manage the latter diseases/conditions. In any case, clinical evidences are still lacking and no studies have been found in the abovementioned database https://clinicaltrials.gov/.

## Conclusion

*Nigella* seeds and extracts have a wide range of bioactivities, which have been demonstrated *in vitro* and *in vivo*. Nonetheless, *N. sativa* is the most studied species probably due to their popularity in folklore medicine. In all cases, future perspectives should be oriented to perform a better characterization of the constituents of the extracts since most studies were only focused on determining the concentration of thymoquinone, but other phytochemicals could be present as in this review more than 80 compounds have been reported. This is also important to remark that bioavailability could affect the way that *Nigella* components act *in vivo*, e.g., some studies administered *Nigella* products orally, while other intravenously.

When referring to clinical trials, it seems that the supplementation of *N. sativa* can exert some effects on diabetes, obesity, and hypertension, among others. Nonetheless, the administration way (seeds, powder, oil, etc.), the dose and the treatment period are highly variable and should be further established from a therapeutic point of view, as well the chemical and phytochemical composition. This should be further study in order to formulate functional ingredients/nutraceuticals to promote the health benefit and how should be added into food. Moreover, besides the pleiotropic health applications of *Nigella*, it can be useful in nanotechnology applications as well as to modulate the activity of other therapeutic agents.

It is important to highlight that the culinary and medicinal use of some *Nigella* seeds as spice, condiment or infusion (e.g*.*, *N. sativa*, *N. damascena*, and *N. arvensis*) suggests that their consumption is safe. The seeds are characterized by a low toxicity degree (Ali and Blunden, 2003) and no serious side effects in clinical trials (Namazi et al., 2018), but more studies were focused on *N. sativa*. In fact, toxicity studies performed in animal models also suggest a wide of margin of safety for using *N. sativa* fixed oil, with high LD_50_ value (28.8 ml of oil/kg BW administered orally to mice) (Zaoui et al., 2002), in powder form (Dollah et al., 2013) and as extracts (Vahdati-Mashhadian et al., 2005).
